# Molecular Dynamics Simulations of Plasma–Antifolate Drug Synergy in Cancer Therapy

**DOI:** 10.3390/biom15060773

**Published:** 2025-05-27

**Authors:** Yanxiong Niu, Tong Zhao, Xiaolong Wang, Ying Sun, Yuantao Zhang

**Affiliations:** School of Electrical Engineering, Shandong University, Ji’nan 250061, China; niuyx@mail.sdu.edu.cn (Y.N.); wangxiaolong@sdu.edu.cn (X.W.); ys2018@sdu.edu.cn (Y.S.); ytzhang@sdu.edu.cn (Y.Z.)

**Keywords:** plasma-assisted cancer therapy, hSLC19A1, 5-MTHF, pemetrexed, molecular dynamics simulation

## Abstract

Reactive oxygen species (ROS) generated by cold atmospheric plasma (CAP) cause irreversible damage to cancer cell DNA, RNA, mitochondria, and antioxidant defense systems, leading to apoptosis. Plasma-induced disruption of the antioxidant defense system of cancer cells by cystine uptake via xC^−^ antiporter has been widely studied, while folate uptake by cancer cells via high expression of hSLC19A1, which generates Nicotinamide Adenine Dinucleotide Phosphate (NADPH) via one-carbon metabolism, is also an important component of the antioxidant defense mechanism of cancer cells. Disrupting folate transport in cancer cells is an important potential pathway for synergizing with pemetrexed (PMX) to induce apoptosis in cancer cells, which is of great research value. In this paper, classical molecular dynamics simulations were employed to study the effect of plasma oxidation of hSLC19A1 on the uptake of 5-Methyltetrahydrofolate (5-MTHF), which is the predominant dietary and circulatory folate, and the antifolate chemotherapeutic agent PMX by cancer cells. The results showed that the channel radius of hSLC19A1 for transporting 5MTHF after oxidation became narrower and the conformation tended to be closed, which was unfavorable for the transport of 5-MTHF; hydrogen bonding and hydrophobic interactions between hSLC19A1 and 5-MTHF decreased, the predicted docking affinity decreased, and the binding energy decreased from −28.023 kcal/mol to −16.866 kcal/mol, while that with PMX was stable around −28 kcal/mol, suggesting that the oxidative modification reduced the binding capacity of hSLC19A1 and 5-MTHF while barely affecting the transport of PMX, which contributed to weakening the antioxidant defense system of cancer cells and synergizing with PMX to induce apoptosis in cancer cells. Our simulations provide theoretical insights for CAP-induced apoptosis in cancer cells at the microscopic level and help promote the further development of cold atmospheric plasma in the field of cancer therapy.

## 1. Introduction

Cancer poses a serious threat to human health, with incidence and mortality rates exhibiting steady annual increases [1]. Conventional methods for treating cancer, such as surgery, radiation therapy, chemotherapy, and hematopoietic cell transplantation, can lead to serious adverse effects during clinical application [2]. Over the course of long-term treatment, cancer cells develop resistance to chemotherapy drugs, limiting the effectiveness of traditional radiation and chemotherapy approaches for cancer treatment. Developing new therapeutic methods to enhance efficacy against tumors is a significant challenge faced in the clinical treatment of cancer. Cold atmospheric plasma treatment for cancer holds great potential and is currently one of the most widely studied applications in the field of plasma biomedicine [3]. Reactive oxygen species (ROS) generated by cold atmospheric plasma (CAP) irreversibly damage DNA, RNA, mitochondria, and antioxidant defense systems of cancer cells, leading to oxidative stress and, ultimately, apoptosis [4]. Researchers have explored plasma treatment for cancer across more than 20 types of cancers, including skin cancer, head and neck cancer, brain cancer, lung cancer, cervical cancer, and leukemia [5,6,7,8,9,10].

Plasma exhibits selectivity toward cancer cells due to the fact that cancer cells have higher internal levels of ROS than healthy cells, which enables them to be able to reach the apoptotic threshold faster under plasma treatment [11]. To counteract oxidative stress, tumor cells upregulate NADPH levels in various ways to generate tolerance to ROS [12], and increasing hSLC19A1 expression on cancer cell membranes, thus upregulating folate-mediated one-carbon metabolism, is one of the main pathways [13]. Folate can also exert direct antioxidant effects by inhibiting lipid peroxidation (LPO) and protecting biological components such as cell membranes or DNA from ROS damage [14]. Folate-mediated antioxidant effects are essential for ROS resistance in tumor cell growth and metastasis [15]. Experimental evidence demonstrates that folate deprivation triggers the overproduction of ROS, leading to cancer cell apoptosis [16], and the knockdown of folate-related genes in hepatocellular carcinoma cells consistently reduces the production of NADPH, which allows for the accumulation of ROS [17]. Ducker et al. [18] reported that cell lines with impaired folate-related enzymes were sensitive to hydrogen peroxide, leading to oxidative stress and inflammation. Ye et al. showed that mitochondrial folate transporter mutations elevate superoxide levels, significantly depleting mitochondrial and cytoplasmic folate levels, impairing antioxidant defenses, and exacerbating mitochondrial dysfunction following tert-butylhydroperoxide (tBH) treatment [19]. Chern et al. cultured hepatocellular carcinoma cells in folate-deficient medium, and observed a dramatic increase in lipid peroxidation indices [20], which resulted in the apoptosis of cancer cells. These studies indicate that the plasma disruption of folate transport in cancer cells can weaken the antioxidant defense system of cancer cells, which is of great scientific importance.

Extensive research has investigated the mechanism by which plasma disrupts cancer cell antioxidant systems. Xinpei Lu et al. found that the liquid-phase active particles generated by plasma acting on cancer cells lead to a decrease in the GSH/GSSG ratio, NADPH/NADP+ ratio, and superoxide dismutase content, and a significant increase in the intracellular ROS content, which in turn causes damage to the intracellular antioxidant system [21]; Bauer’s study showed that cold atmospheric plasma depletes intracellular glutathione in cancer cells, thereby eliminating cellular protection against lipid peroxidation [22]; Bekeschus et al. found that high expression of xC^−^ antiporter increased tumor cell resistance to plasma, and silencing its associated genes restored tumor cell sensitivity to plasma [23]; Moniruzzaman et al. also proposed combining plasma therapy with xC^−^ inhibitors for the treatment of cancer [24]; and Yusupov et al. investigated the effect of plasma-induced mutations of Cys residues on xC^−^ antiporter on the transport of cystine by molecular dynamics simulations [25]. These studies predominantly focused on the xC^−^ antiporter pathway, while folate-mediated NADPH metabolism is also an important part of the antioxidant system in cancer cells, and the plasma treatment of cancer cells will make contact with the folate transporter hSLC19A1 in the early stage, which is expressed in a large amount on the membrane of the cancer cells [26], and it is hypothesized that CAP-induced oxidation of folate transporter will play an important role in weakening the antioxidant defense mechanism of the cells, which is of great significance for understanding the molecular mechanism of apoptosis in cancer cells by plasma.

5-Methyltetrahydrofolate (5-MTHF) is the predominant dietary and circulatory folate, and is transported into cancer cells via hSLC19A1, which is highly expressed in the cancer cell membrane. At the same time, it is important to note that hSLC19A1 is the target of the antifolate chemotherapeutic agent pemetrexed (PMX), and thus the effect of plasma oxidation on the transport of 5-MTHF by hSLC19A1 as well as PMX needs to be considered at the same time. hSLC19A1’s transport of 5-MTHF and PMX remains challenging to observe experimentally, but molecular dynamics simulations offer a powerful tool to reveal the microscopic mechanisms of life processes and explain the problems that are difficult to observe experimentally at the molecular level, which have been widely used in the biomedical field. Rezaei et al. used molecular dynamics simulations to study the effect of oxidation of VDAC 1 by cold atmospheric plasma on the uptake of pyruvate by VDAC 1, showing that plasma can destroy the antioxidant system of cancer cells [27]; Yusupov et al. also used molecular dynamics simulations to study the effect of residues oxidation on cystine transport [25]. Therefore, in this paper, the classical molecular dynamics software GROMACS (version: 2023.2) was chosen for the subsequent study.

In this paper, the effect of CAP-induced oxidation of hSLC19A1 on the transport of 5-MTHF and PMX was simulated and investigated based on classical molecular dynamics simulations. Structural alterations of hSLC19A1 were analyzed via metrics including channel radius, secondary structure, RMSD and RMSF; the effects of plasma oxidation on the transport of 5-MTHF and PMX were compared by analyzing the hydrophobic interactions and hydrogen bonds between hSLC19A1/hSLC19A1_OX_ and 5-MTHF/PMX, performing molecular docking and calculating the binding free energy. The results show that the channel radius of hSLC19A1 transporting 5-MTHF narrows at the bottom of the channel at 4.5–6 nm on the *z*-axis under plasma oxidation; secondary structure stability decreases, and the flexibility changes of Cys30,33 and Met130 residues lead to the inward tightening of the channel entrance; hydrogen bonds number formed with 5-MTHF were reduced by an average of 0.71 per frame and hydrophilic interaction was weakened; and the repulsion of the free energy of polar solvation leads to a 40% decrease in the binding energy with 5-MTHF, while there is no significant change on the structure of hSLC19A1 when bound to PMX or on the interaction between hSLC19A1 and PMX. This study demonstrates that CAP selectively impairs the ability of hSLC19A1 to transport 5-MTHF while preserving PMX transport, and reduces the uptake of folate by cancer cells without hindering the antifolate therapy, which can help to weaken the antioxidant defense system of cancer cells and elevate intracellular ROS levels, thus enhancing its cytotoxic selectivity toward cancer cells, and prompting cancer cell apoptosis. The results of this paper contribute to the further development of cold atmospheric plasma in the biomedical field.

## 2. Methods

### 2.1. Protein Modeling

**Figure 1:**
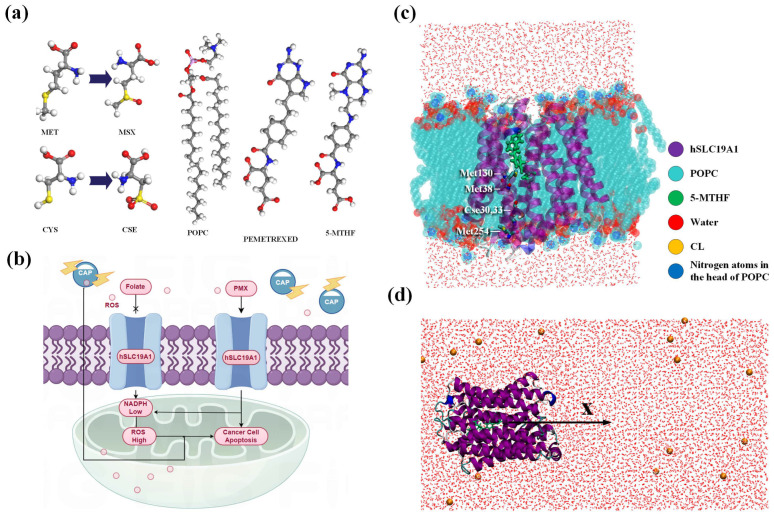
(**a**) Molecular structures of POPC, PMX, 5-MTHF, Met, and Cys; (**b**) schematic diagram of plasma synergizes with PMX to induce apoptosis in cancer cells; (**c**) model of hSLC19A1 embedded in POPC cell membrane; (**d**) umbrella sampling model.

The original hSLC19A1 conformation interacting with 5-MTHF and PMX was obtained from the RSCB Protein Data Bank (https://www.rcsb.org/, accessed on 9 October 2024) (8GOE, 8GOF). hSLC19A1 consists of more than 400 residues and has a predominantly α-helical structure, with a distinct substrate-binding pocket in the center spanning both ends of the phospholipid bilayer, as shown in Figure 1c. In order to obtain the oxidized hSLC19A1 model, oxidative modification of specific residues was applied on VIENNA-PTM2.0 [28,29,30]. It was reported that the sulfur-containing amino acids Met and Cys are highly reactive and susceptible to plasma oxidation, with Met being mainly oxidized to (Met+O)H+ after five minutes and Cys being irreversibly oxidized to (Cys+3O)H+ in the presence of active particles, such as hydroxyl groups and hydrogen peroxide [31,32,33,34]. In order to investigate the specific oxidative modification sites of the plasma active particles, a solvent accessible surface area (SASA) analysis was carried out; residues with larger SASA have a higher probability of contact with the plasma and a higher probability of being oxidized. The results of the analyses are shown in Table 1, and it is clear that the SASA of Met119 and 122 and Cys365 and 396 is much lower than that of Met38, 130, and 254 and Cys30 and 33, suggesting that the former are buried inside the protein and have little access to the active particles. Therefore, the five residues Cys30 and Cys33, Met38, 130, and 254 should be considered for modification because they have the highest probability of being oxidized. All modified residues have been pre-energy-minimized using the GROMOS54A7 force fields before being incorporated into hSLC19A1 to optimize the geometry and energy of the oxidized hSLC19A1. In total, 1500 minimization steps were performed in vacuum with a maximum force convergence threshold of 1.0 kJ/mol/nm. A cut-off range of 1.4 nm was used for both the van-der-Waals and Coulomb interactions [28]. Cys30, 33, and Met38 are located on the transmembrane domain 1a of hSLC19A1, and Met130 is located at the bottom of the binding pocket on TM4 where hSLC19A1 binds to the benzoyl portion of 5-MTHF/PMX, which is important for protein–ligand binding, while Met254 is located on the end of intracellular loop IL6-7, farther away from the binding pocket, as shown in Figure 1c [35]. The Solvent accessible surface area (SASA), polarity (charge), and other properties of Met and Cys changed significantly after plasma modification, with the average polarity of Met increasing from 2.62 to 4.27, and Cys changing from electrically neutral to one that is negatively charged [30]. Thus, plasma oxidation of the corresponding residues may alter the movement of transmembrane domain 1a as well as the substrate-binding pocket environment, thereby having an important impact on the transport of 5-MTHF and PMX by hSLC19A1.

### 2.2. Simulation Details

To investigate the effect of plasma on the transport of folate, as well as PMX by hSLC19A1, a total of four systems were created, including two native systems, as well as the oxidized proteins transporting 5-MTHF and PMX, using 19A1 as an abbreviation for hSLC19A1, named 19A1-5MTHF, 19A1ox-5MTHF, 19A1- PMX, and 19A1ox-PMX, as shown in Table 2. The topologies of the ligand molecules were generated in the Automated Topology Builder (https://atb.uq.edu.au/, accessed on 9 October 2024) [36,37] and introduced into the overall topology file. Studies have shown that lipid molecules play an active role in regulating the structure and function of membrane proteins [38,39], so the protein was embedded in a phospholipid bilayer composed of 128 POPC molecules using the membed method, so that the protein was first scaled down to 0.1 times its original size in the xy-direction, and then gradually swelled to its normal size during the simulation in 1000 steps, so that the phospholipid was in close contact with the protein. A cubic box with dimensions of 7.15 × 7.15 × 8.95 nm^3^ was solvated by adding water molecules and NaCl, followed by energy-minimization using the conjugate gradient algorithm. Positional constraints were then imposed on the hSLC19A1 backbone and ligand weight atoms, and constrained molecular dynamics simulation of 500 ps was carried out using a V-rescale thermal bath in the nvt ensemble, with a time step of 0.001 ps. Then, npt simulation was carried out using a V-rescale thermal bath and a C-rescale pressure bath, allowing the system to gradually reach equilibrium without strongly disturbing its original conformation [40]. Subsequently, positional restraints were removed, the temperature was maintained at 298.15 K in order to facilitate comparison with the experimental binding data obtained afterwards, and the pressure was maintained at 1 atmosphere for a final equilibrium simulation of 100 ns. Electrostatic interactions were treated using the PME method. Periodic boundary conditions were applied in all three dimensions. The simulated time step is 2 fs and the LINCS algorithm is applied to maintain the rigidity of the molecular bonds. The TIP3P water model was used to solvate all simulated systems. All simulations were performed using the GROMACS (Vision: 2023.2) software package with the GROMOS54A7 force field containing non-standard residue descriptions provided on the PTM 2.0 website (http://vienna-ptm.univie.ac.at/, accessed on 18 October 2024), and a description of the phospholipid molecules was added to the force field. All molecular visualizations were performed using VMD (Vision: 1.9.3) [41]. In this paper, the simulation is repeated more than three times for each system to avoid errors arising from randomness [42,43,44,45].

### 2.3. Free Energy Calculations

In order to calculate the effect of plasma oxidation on the binding energy between hSLC19A1 and the ligand and to analyze the contribution made by each residue to the binding energy, we also calculated the binding energy of all systems using the molecular mechanics Poisson–Boltzmann surface area (MMPBSA) method [46]. The MMPBSA method integrates three computational methods: the molecular mechanics force field (MM), the Poisson–Boltzmann equation, and the solvent-accessible surface area (SASA), which is widely used for the calculation of interaction energies between biomolecules, which allows for a comparison of the relative energetic contribution of each residue to the total binding energy. Umbrella sampling was also conducted in order to more precisely show the effect of plasma oxidation on the binding of 5-MTHF and hSLC19A1. The protein and ligand were placed in a rectangular box elongated by 7.0 nm in the *x*-axis direction for pulling, and the constructed model was equilibrated in an NPT ensemble for 20 ns for Steered molecular dynamics simulations where the ligand was slowly pulled along the *x*-axis at a rate of 0.01 nm/ps. The ligand was slowly pulled along the *x*-axis direction at 0.01 nm/ps with a harmonic bias force constant of 1000 kJ mol^−1^nm^−2^. After the Steered molecular dynamics simulation, a series of windows were extracted at intervals of 0.1 nm along the *x*-axis, for a total of 50 windows, and batch-independent simulations were subsequently carried out for each of the windows. Following the stretching simulations, a series of windows were extracted along the *x*-axis, followed by batch-independent simulations for each window to obtain a single free energy of dissociation, and finally the Weighted Histogram Analysis Method (WHAM) was applied to calculate the binding free energy of 5-MTHF/PMX dissociated from the hSLC19A1/hSLC19A1ox binding site.

## 3. Results and Discussion

### 3.1. Changes in Protein Structure

**Figure 2:**
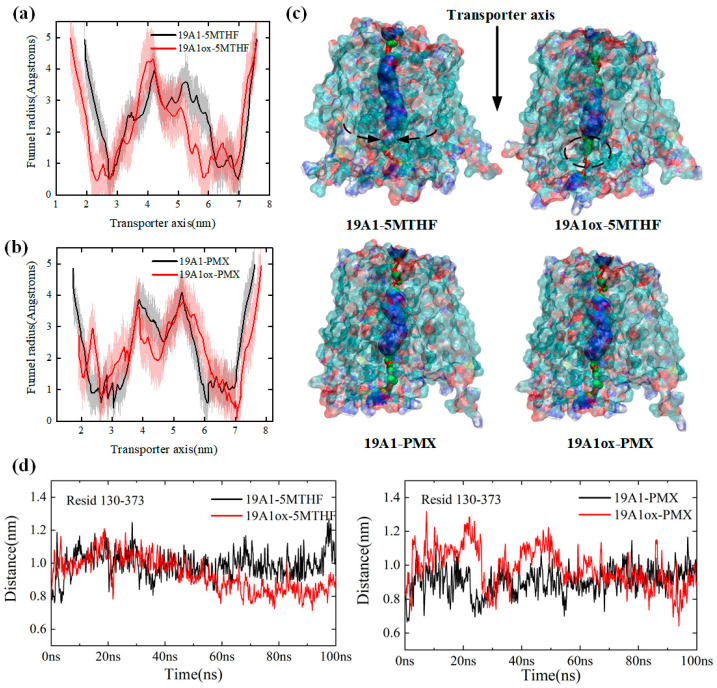
(**a**) Comparison of channel radii of hSLC19A1-5MTHF before and after plasma oxidative modification; (**b**) comparison of channel radii of hSLC19A1-PMX before and after plasma oxidative modification; (**c**) schematic representation of the hSLC19A1 transmembrane channels in the four systems; (**d**) variation of distance between Met/Msx130 and Arg373.

In this section, the structural changes of the protein after oxidation by plasma are analyzed. Firstly, the transmembrane channel radii of native and plasma-oxidized hSLC19A1 were calculated after equilibrium, as shown in Figure 2a,b, to explain the effect of plasma oxidized modifications on the hSLC19A1 monomer structure and transmembrane transport function [47].

hSLC19A1 consists of 12 transmembrane domains, and each transmembrane domain is abbreviated as TM. In the green region of Figure 2c, TM 1, TM 2, TM 7, and TM 8 are tightly coupled with each other, creating the narrowest constriction of the monomeric pore from the top, with an average radius of around 0.6 Å before oxidation, which is reduced to 0.5 Å in the 19A1ox-5MTHF system. The blue region is the main pocket for the binding of hSLC19A1 and ligand, with a total length of about 35 Å. At the end of the channel, TM 4, and TM 5, as well as TM 10 and TM 11, dissociate to form the entrance to a large polar cavity within the transporter. The entire monomeric channel extends about 5.5 nm in length, longer than the thickness of the phospholipid bilayer (about 5 nm), and is therefore capable of transmembrane transport.

The red curve in Figure 2a illustrates the channel radius of hSLC19A1_OX_-5MTHF along the *z*-axis. Compared with the native system, the plasma-oxidized hSLC19A1 channel narrows significantly upon translocation of 5-MTHF, with the average channel radius narrowing from 2.384 Å in the native system to 2.173 Å. The most significant change in the radius of the substrate-binding pocket was observed between 3 nm and 6.2 nm along the *z*-axis, with the pocket length decreasing from 3.2 nm to approximately 2.8 nm. The 5.9 nm from the z axis at the channel inlet radius decreased from 1.3 nm to 0.56 nm, suggesting that the outward conformation of hSLC19A1 tends to be closed, limiting substrate entry. Met130 is located at the bottom of the pocket where hSLC19A1 binds to the benzoyl group of 5-MTHF/PMX, and the distance between it and Arg373 on the channel’s opposite side is able to reflect the radius of the protein’s channel at 5–6 nm. Figure 2d shows the variation of the distance between Met/Msx130 and Arg373 for the four systems; it can be seen that the distance between Msx130 and Arg373 in the 19A1ox-5MTHF system decreased significantly between 20 ns and 60 ns, while it fluctuated around 0.8 nm after 60 ns, which is about 0.2 nm lower than that before oxidation, which verifies the results of the channel radius analysis, suggesting that the attraction between Met/Msx 130 and Arg 373 resulted in the decrease in channel radius, which may be the result of oxidized Met130 the result of increased polarity. The narrow channel would hinder folate uptake by cancer cells and may also adversely affect the binding affinity of the protein to folate. In contrast, there was no significant change in the radius of the PMX substrate-binding pocket in Figure 2c, and the distance between Msx130 and Arg373 was not shortened in the 19A1ox-PMX system, reaching equilibrium after 60 ns and fluctuating around 1.0 nm, suggesting that the oxidation of hSLC19A1 by CAP did not structurally affect PMX transport while hindering folate uptake into the cancer cells, offering a mechanistic basis for plasma-conjugated antifolate therapies targeting cancer cells.

**Figure 3:**
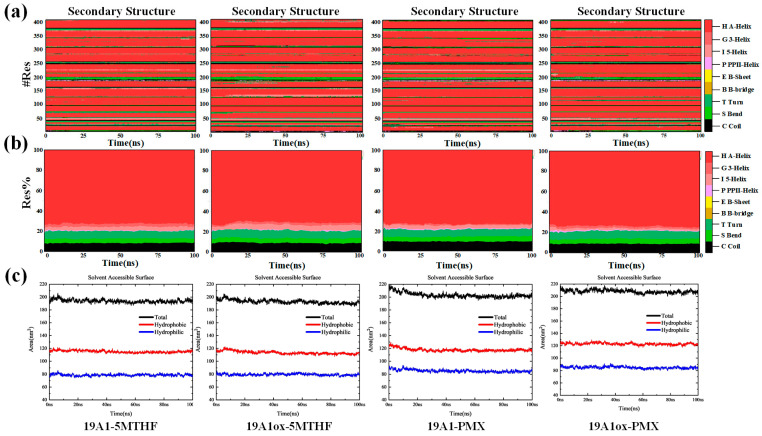
(**a**) Changes in the secondary structure of individual residues during the simulation of the four systems. (**b**) Proportion of different secondary structures in the total. (**c**) Changes in solvent-accessible surface area during simulation.

The secondary structure was analyzed to obtain the evolution in the secondary structure of each residue of hSLC19A1 and the proportion of the different secondary structures in it during the simulation of the four systems, as shown in Figure 3. A-Helix is the most common secondary structure and the main component of hSLC19A1. In 19A1-5MTHF, 19A1-PMX, and 19A1ox-PMX, the content of A-Helix was stable at about 73%, indicating that the transport structure of the protein was not significantly affected. In contrast, the percentage of A-Helix in the 19A1ox-5MTHF system decreased by about 3% after 20 ns, suggesting that the oxidized hSLC19A1 may have a reduced ability to transport 5-MTHF. It was observed that residues near Cse30, Cse33, and Met38 in the 19A1_ox_-5MTHF system tended to shift to T-turn, the appearance of which, as a short-range folding unit, may disrupt the continuity of the A-Helix, thereby affecting the aperture size of the transmembrane channel as well as the conformation of the substrate-binding pocket. In order to further assess the equilibrium of the system, SASA analysis of the protein was performed, as shown in Figure 3c. It can be seen that since hSLC19A1 is a membrane protein, most of the structure is embedded inside the hydrophobic phospholipid bilayer, and the hydrophobic SASA is higher than hydrophilic SASA. In all four systems, SASA reached equilibrium within 20 ns, demonstrating that the simulations reached stability. For the 19A1ox-5MTHF system, the hydrophobic SASA decreased by about 3.5 nm^2^ after the simulation while the hydrophilic SASA remained unchanged, which is also consistent with the result that the overall conformation of 19A1ox-5MTHF tends to be inwardly closed, as shown in the following section, whereas the hydrophilic SASA of 19A1ox-PMX did not change much, with the overall conformation being stable.

Furthermore, residues near Met130 shifted from A-Helix shifts to S bend and I 5-helix, suggesting a possible enhancement of flexibility in this region, which arises from plasma oxidation of the corresponding residues. Overall, the oxidative modification specificity of the plasma affects the helix stability of the 5-MTHF binding region.

**Figure 4:**
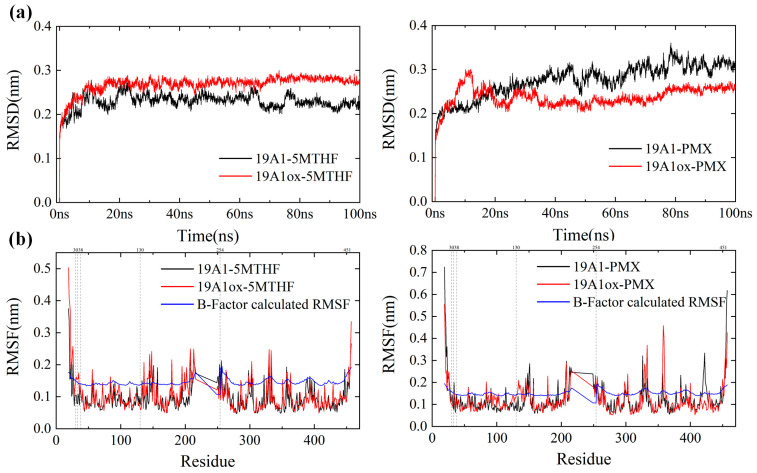
(**a**) RMSD changes during system simulation. (**b**) RMSF of the protein backbone of the four systems against the RMSF calculated by B-factor.

Root mean square deviation (RMSD) is a widely used measure of protein structural deviation, and the system can be considered to have reached a steady state when the overall RMSD value stabilizes within a narrow range of values over time. Figure 4a shows the variation of the protein backbone RMSD values of the four systems over the 100 ns simulation. The RMSD of native system 19A1-5MTHF fluctuated greatly before 10 ns of simulation and reached stability after 10 ns, maintaining fluctuation around 0.23 nm, which is consistent with the trend of the secondary structure, indicating that the native system was close to reaching equilibrium after 10 ns, and the deviation from the experimentally measured protein structure was within a reasonable range, reflecting that the simulation parameters were reasonable. In contrast, after being oxidized by plasma, the RMSD of the 19A1ox-5MTHF system took a longer time to reach stability, and gradually reached equilibrium after 17 ns, after which it maintained, fluctuating around 0.3 nm, and the RMSD at the time of the stabilization was higher than that of the native system, which indicates that the protein structure changed, reflecting that the plasma modified hSLC19A1, which also resulted in a weakening of the binding ability to 5-MTHF. In contrast, the RMSD of 19A1_ox_-PMX, although showing a rapid rise at 10 ns, stabilized to near 0.2 after 17 ns, and was lower than that of the native system at the later stage of the simulation. This suggests that the presence of PMX keeps the structure of the oxidized hSLC19A1 stable and is not overly disturbed by the oxidative modifications of the plasma.

**Figure 5:**
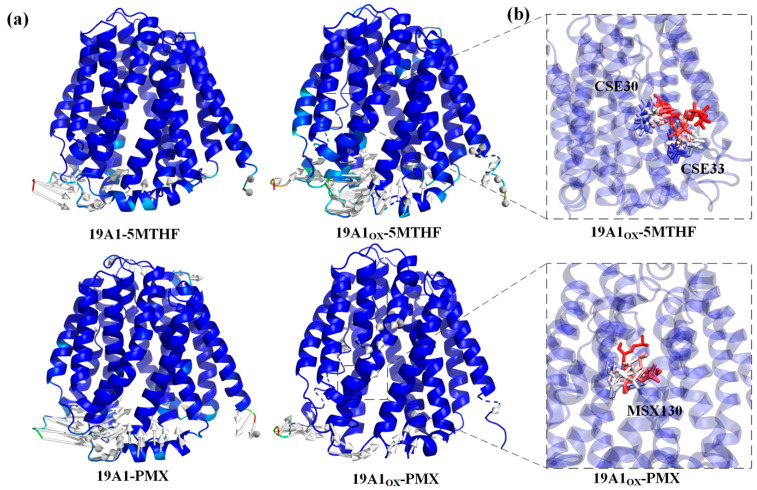
(**a**) Comparison of protein structural differences before and after simulation. (**b**) Overlay of residue motions over time trajectories with red frames ahead in time and blue frames behind in time.

The Cα root mean square fluctuation (RMSF) quantifies the atomic-level conformational flexibility of each residue in a molecular dynamics (MD) trajectory, and the part of the region with higher flexibility has more fluctuating protein motion and has larger RMSF values while the region with lower flexibility is more stable. Figure 4b shows the RMSF comparison between the oxidized system and the native system. The trends of RMSF for all four systems are consistent with the RMSF calculated from the B-Factor of the initial conformation of hSLC19A1 (blue curve in Figure 4b), and thus the current simulation parameters can be considered reasonable. From the figure, it can be seen that the RMSF of the protein in 19A1ox-5MTHF is obviously increased at residues Cys30, 33, and Met38. Cys30 and 33 and Met 38 are located on transmembrane domain 1a. Simulated snapshots in Figure 5 reveal that the transmembrane domain 1a of 19A1_ox_ is inwardly tightened, and the shift of the secondary structure of Cys33 from A-Helix to T-turn hinders the entry of folate into the binding pocket, which is in agreement with the results of the secondary structure analysis. In contrast, the change of protein flexibility in the 19A1_ox_-PMX system mainly occurred near the Met130 residue, which is located on TM4, and it can be seen in Figure 5 that the increase in its flexibility led to the outward opening of the TM4 end, which was also the reason for the slight increase in the radius at the entrance of the channel, and the PMX-binding region remained structurally stable without observable conformational perturbations. It should be noted that resid 217–248 are missing from the sequence due to the difficulty of determining its dynamic structure, which does not affect the calculations since it is far from the binding pocket [35].

Overall, the oxidative modification of CAP narrows the radius of the channel of hSLC19A1 upon transporting 5-MTHF, disrupts its secondary structure, and alters the conformational flexibility of critical residues, which impedes 5-MTHF entry into cancer cells, thereby impairing one-carbon metabolism, and weakening their antioxidant defense systems. Meanwhile, PMX exhibits strong affinity binding to hSLC19A1, maintaining structural stability even under plasma-induced oxidative conditions, indicating that the oxidative modification of hSLC19A1 by plasma did not structurally affect PMX transport, and it could synergize with PMX for antifolate therapy, which proposed a new strategy for cancer treatment.

### 3.2. Analysis of Changes in Protein–Ligand Interactions

In order to further investigate the effect of plasma oxidation of hSLC19A1 on its transport function, it is necessary to analyze the changes in hydrogen bonds and hydrophobic interactions and binding energies between 19A1 and 5-MTHF as well as PMX before and after plasma modification. For this purpose, hydrogen bonds and hydrophobic interactions were analyzed. The binding free energy between 19A1 and the ligand was also calculated using the MMPBSA method as well as umbrella sampling, and molecular docking was also carried out to assess the binding affinity.

**Figure 6:**
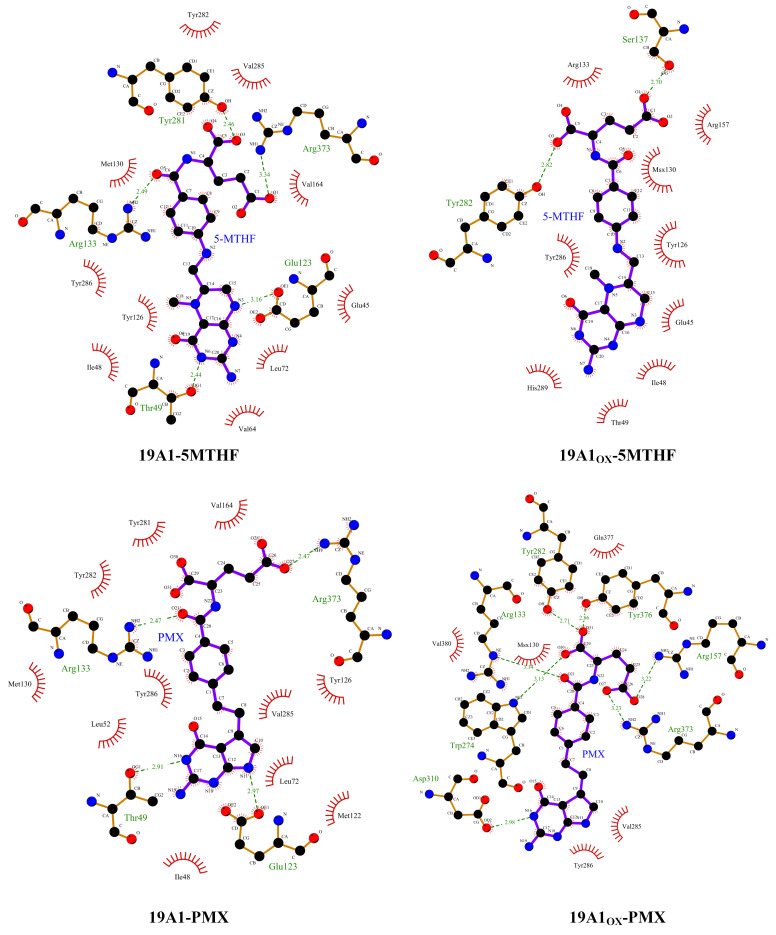
Schematic representation of the hydrophobic interaction and hydrogen bonds. the red eyelash pattern represents hydrophobic interactions and the green dashed line represents hydrogen bonding.

**Figure 7:**
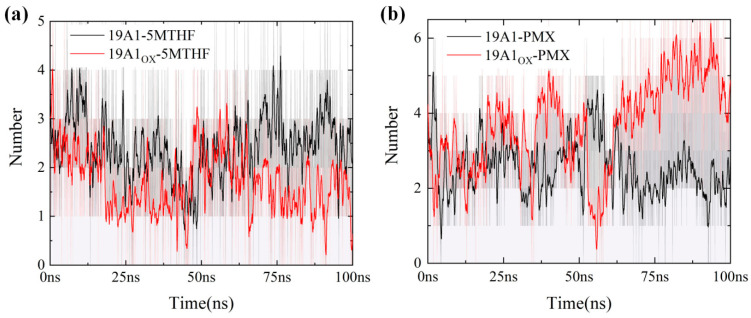
Number of hydrogen bonds in 100 ns for six systems (averaged over 20 adjacent frames): (**a**) 5MTHF system; (**b**) PMX system.

The phenomenon of hydrophobic groups aggregating close to each other to avoid water is known as the hydrophobic interaction and is the main driver of protein folding. In this paper, we used ligplot [48,49] to study the formation of hydrogen bonds and hydrophobic interactions between proteins and ligands after 100 ns simulation, as shown in Figure 6, and the probability and position of the appearance of each hydrogen bond within 100 ns were counted using VMD in Figure 7 and Table 3. The donor-acceptor distance was set to be 3.5 Å and the angular cut-off was 43.1°, corresponding to 30° for the GROMACS calculation of hydrogen bonding. As can be seen in Figure 6, in the native system, the pterin portion of 5-MTHF occupies the uppermost negatively charged pocket of the channel, forming hydrophobic contacts with Ile48 of TM 1, Leu72 of TM 2, and Tyr 126 of TM 4. The glutamate portion faces the inside of the cell and is close to the positively charged region of the protein. The γ-carboxylic acid group interacts with Arg133 of TM 4 and Arg373 of TM 10, the β-carboxylic acid group interacts with Tyr281/Tyr282 of TM 7 and Gln377 of TM 10, and the carbonyl group interacts with Arg133 of TM 4. The benzoyl group partially bridges the pterin- and glutamate-mediated interactions and forms hydrophobic interactions with Tyr126/Met130 of TM 4 and Val285/Tyr286 of TM 7. Upon oxidation, the hydrogen bonds formed by 5-MTHF with Tyr281, as well as the hydrophobic interactions, were reduced, and Table 3 shows that the number of hydrogen bonds formed per frame in the simulations decreased from 0.9895 to 0.3628, and the number of hydrogen bonds formed with Arg133 decreased from 0.2724 to 0.0705. Both residues are important for binding to the glutamate portion of the 5-MTHF tail before oxidation [50], and the reduction of hydrogen bonding with 5-MTHF is an important reason for the decrease in the binding of 5-MTHF to 19A1, suggesting that plasma oxidation has caused damage to the charge environment at the bottom of the substrate binding pocket of hSLC19A1. Meanwhile, the glutamate portion of 5-MTHF formed hydrophobic interactions with only two residues, Arg 133 and Arg 157, in the oxidized hSLC19A1, suggesting that the binding ability between the glutamate portion of 5-MTHF and the hydrophobic pocket between the proteins was also weakened.

The pyrimidine-pyrrole, benzoyl, and glutamate portions of PMX form contacts with hSLC19A1 similar to those of 5-MTHF, highlighting a conserved substrate recognition mechanism for folate and antifolate substrates. However, in addition to these conserved functions, 5-MTHF and PMX still exhibit significant differences in their detailed conformations. Specifically, the planes of the pterin ring in 5-MTHF and the pyrimidine-pyrrole ring in PMX exhibit relative rotation and displacement, which in turn induces adjustments in the benzoyl and glutamate portions, leading to differences in hSLC19A1 binding to 5-MTHF and PMX before and after oxidation. After oxidation, the formation of hydrogen bonds between 19A1_OX_ and Tyr282, Tyr376, and Gln377 increased, with an average of one more hydrogen bond formed per frame, which made the binding of PMX to hSLC19A1 somewhat more robust.

**Figure 8:**
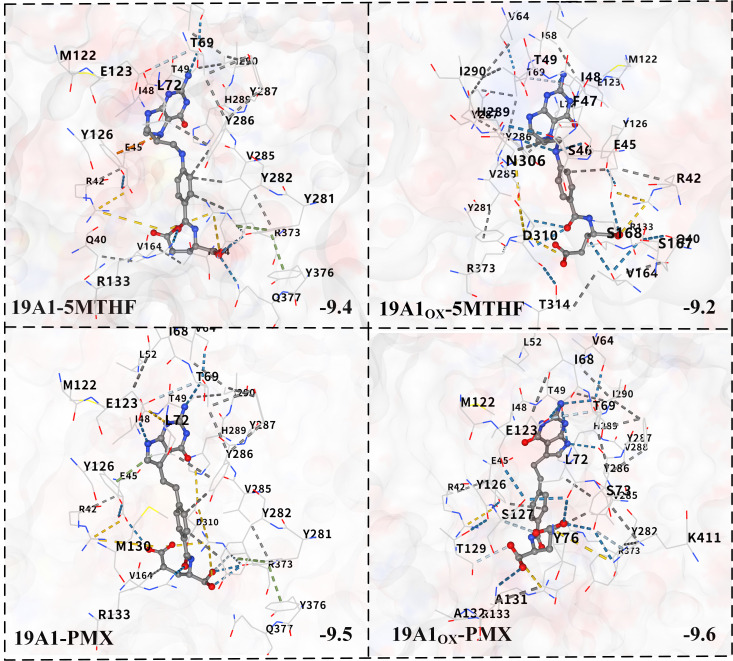
Schematic diagram of molecular docking results. Blue dashed lines represent predicted hydrogen bonds, grey dashed lines represent predicted hydrophobic interactions, and yellow dashed lines represent predicted ionic interactions.

Molecular docking was performed based on the vina algorithm, as shown in Figure 8 [51]. The vina score reflects the strength of affinity between the protein and the ligand, and at below −9, it indicates that the two are able to bind stably. The vina scores of both native systems were below −9, indicating that both 5-MTHF and PMX are ligands with strong binding ability to hSLC19A1. PMX had a vina score as high as −9.5, which was stronger than 5-MTHF, and thus, PMX was preferentially transported by hSLC19A1. Upon plasma oxidation, the predicted binding affinity of 19A1ox-5MTHF was reduced to −9.2, whereas the score of 19A1ox-PMX increased to −9.6, which is consistent with the results of the protein structural analyses and protein–ligand interaction analyses, indicating that the affinity of hSLC19A1 for PMX was significantly higher than that of 5-MTHF after plasma oxidation.

To further investigate the binding capacity between hSLC19A1 and 5-MTHF and PMX under plasma action, we calculated the binding free energies broken down to individual residues using the molecular mechanics-Poisson Boltzmann surface area (MMPBSA) method, which is widely used in the calculation of binding free energies. The overall protein–ligand binding free energy contributions to each branch are shown in Table 4. In the table, dG is the binding free energy, the MM energy is mainly the internal interaction energy, which contains two items, the Coulomb interaction (COU) and the van der Waals interaction (VDW), and the PBSA energy is used to describe the solvation free energy between the protein and the ligand, i.e., their contribution to the stability and solvation free energies in the solvent.

**Figure 9:**
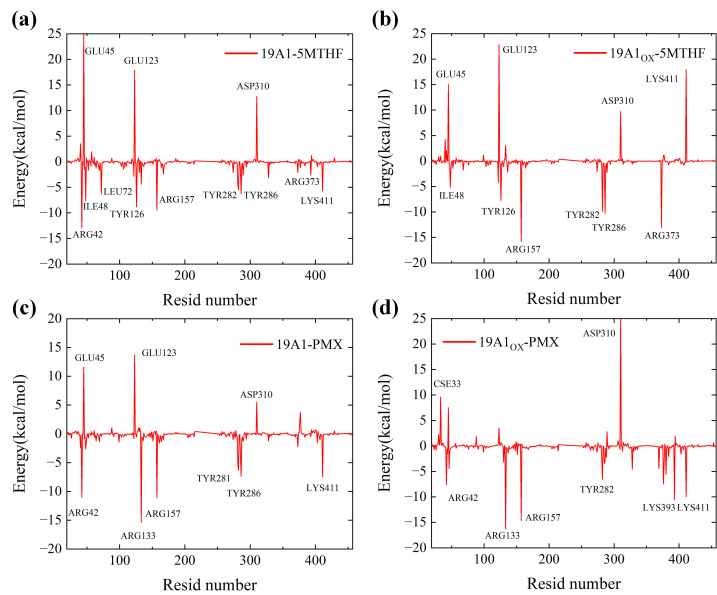
Residue energy contribution diagrams: (**a**) 19A1-5MTHF system; (**b**) 19A1ox-5MTHF system; (**c**) 19A1-PMX system; (**d**) 19A1ox-PMX system.

The binding energy of the native 19A1-5MTHF system was calculated by MMPBSA to be around −28 kcal/mol, which is ‘very strong’, and the binding energy of the native PMX system is slightly higher than that of the 5-MTHF system, which is consistent with the results of molecular docking and protein structure analysis. After plasma oxidation, the Coulomb interaction between 19A1_OX_ and 5-MTHF is slightly enhanced, and the van der Waals hydrophobic interaction does not change much. The repulsive force of the polar solvation energy was significantly increased, indicating that the disruption of the polar environment of the 5-MTHF binding pocket after plasma oxidation of 19A1 counteracted the enhancement of MM, and the final dG was significantly decreased to −16.866 KJ/mol, which is a 40% reduction. In the PMX system, on the other hand, the repulsive force of the polar solvation energy was reduced, which was the main manifestation of the difference between the folate and PMX transporter of hSLC19A1 after plasma oxidation, and the conformation of 5-MTHF and PMX showed relative rotation and shift, with the binding sites not being exactly the same as the root cause of this difference. In order to analyze the contribution of individual residues to the change in binding free energy in more depth, the residue energy contributions were plotted as shown in Figure 9, and the binding energies of several important residues are listed in Table 5.

In two 5MTHF systems, the binding free energy of Arg42 mutated from −12.945 kcal/mol to +2.088 kcal/mol, suggesting that plasma oxidation resulted in a shift from a strong binding contribution to a slight repulsion of this residue. This mainly stems from the significant enhancement of the polar solvation energy (PB) (+14.772 kJ/mol in 19A1-5MTHF and +37.547 kJ/mol in 19A1ox-5MTHF), which may be attributed to the stretching of the side chain of Arg42 after plasma oxidation, which partially exposes the originally buried charged group to the solvent, resulting in the need to overcome a stronger solvation. The change in binding energy of Arg133 also followed the same trend as Arg42, and both residues were located near the negatively charged glutamate portion of 5-MTHF, suggesting that the positively charged pocket of the protein was disrupted in the oxidized system.

Lys411 contributes as much as −5.756 kJ/mol to the native system, and is an important residue in hSLC19A1’s recognition of 5-MTHF, whose polar solvation energy also increased from 8.618 kJ/mol to 31.821 kJ/mol, shifting from strong binding to heavy repulsion. The binding energy of Tyr281 was reduced from −4.430 kJ/mol to −0.925 kJ/mol, in which the MM energy was reduced from −16.222 to −8.428 kJ/mol, and the coulombic interaction was significantly reduced, with some reduction in van der Waals hydrophobic interactions, which is consistent with the analysis of the reduction in hydrogen bonding and hydrophobic interactions formed by Tyr281, as analyzed above. In contrast, in the two PMX systems, the binding energies of important binding residues such as Arg133, Tyr282, and Lys411 remain stable, although the mutation of Cys33 led to an increase in repulsive force from 0.47 kJ/mol to 9.619 kJ/mol. The overall polar solvation energy of repulsion also increased, but the increase in MM energy resulted in no decrease in the overall binding energy. Differences in the intermolecular interaction energies of hSLC19A1 bound to 5-MTHF and PMX after oxidation by plasma, as well as contributions specific to each residue, were revealed by residue free energy decomposition.

**Figure 10:**
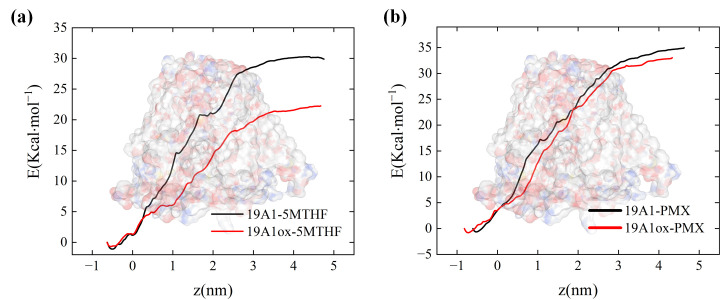
Binding free energy calculated by umbrella sampling: (**a**) natural and oxidized hSLC19A1 with 5-MTHF; (**b**) natural and oxidized hSLC19A1 with PMX.

Figure 10 quantifies the binding free energies of 5-MTHF, as well as PMX, to natural and oxidized hSLC19A1. It is clear from the figure that the binding free energies of 5-MTHF with natural and oxidized channels are 29.88 kcal/mol and 22.25 kcal/mol, respectively. This suggests that under plasma oxidation, the binding between 5-MTHF and hSLC19A1 is weakened, and the ability of the cancer cells to transport folate is reduced, thus hindering the one-carbon metabolic process of the cancer cells and destroying the antioxidant system of cancer cells. In contrast, the decrease in binding energy of PMX was extremely small; 34.94 kcal/mol before oxidizing the protein and 33.04 kcal/mol after oxidation. From the previous analysis, this is due to the rotational and fine-tuning relationship between the conformation of PMX and 5-MTHF. Since PMX would compete with folate for the binding site, the affinity of PMX after plasma oxidation of hSLC19A1 was much higher than that of 5-MTHF, and it would significantly reduce the folate uptake of the cancer cells when transporting PMX into the cancer cells, which indicated that the plasma could synergize with PMX to work together to disrupt the antioxidant system of the cancer cells, and synergize with the plasma to further destroy the key intracellular components.

## 4. Conclusions

In this paper, the effect of cold atmospheric plasma (CAP) oxidation on the uptake of 5-MTHF by cancer cells via hSLC19A1 was explored at the molecular level through classical molecular dynamics simulations, and the interference of cold atmospheric plasma on the transport of the antifolate chemotherapeutic drug pemetrexed (PMX) while affecting the folate transport in cancer cells was considered. The results showed that plasma did not impede the uptake of the antifolate chemotherapeutic drug PMX by hSLC19A1 while disrupting its interaction with 5-MTHF. This opens new avenues to disrupt the antioxidant pathway in cancer cells.

We investigated both hSLC19A1 structural changes and changes in the interaction between hSLC191A1 and 5-MTHF/PMX. The results of the structural study showed that under plasma oxidation, the channel radius of hSLC19A1 upon transporting 5-MTHF narrowed at the bottom of the channel at 4.5 to 6 nm on the z axis, and the overall channel length was shortened; the stability of the secondary structure was reduced, and the secondary structures of Cys30 and 33 and Met130 shifted from A-Helix to structures such as T-turn and S bend. Furthermore, increased flexibility led to inward tightening of the channel entrance. Protein–ligand interaction analyses indicate that the hydrogen bonds formed between hSLC19A1 and 5-MTHF upon oxidation are reduced by an average of 0.71 per frame, followed by a significant reduction in the hydrophobic interaction formed by the glutamate portion of 5-MTHF; molecular docking showed that the predicted affinity of hSLC19A1 with 5-MTHF decreased after oxidative modification; and MMPBSA analysis showed that the repulsion of the free energy of non-polar solvation led to a 40% decrease in the binding energy of hSLC19A1 with 5-MTHF; in contrast, plasma oxidation did not significantly affect the structure of hSLC19A1 when bound to PMX or the interaction between hSLC19A1 and PMX. This study demonstrates that plasma oxidation has no significant effect on the structure of hSLC19A1 when bounded with PMX and the interaction between hSLC19A1 and PMX. This study demonstrates that plasma can reduce the binding capacity of hSLC19A1 to 5-MTHF without disrupting the transport of PMX. Disrupting the uptake of folate by cancer cells while combining with antifolate chemotherapeutic agents can help weaken the antioxidant defense system of cancer cells, increase the level of ROS in cancer cells, and promote the apoptosis in cancer cells.

Our simulations investigated the potential pathways by which plasma disrupts the antioxidant system of cancer cells and considered the possibility of synergistic treatment in combination with conventional antifolate chemotherapeutic agents, explored the intrinsic microscopic mechanisms, provided atomic-level insights into the mechanism of the integrated effect on cancer cells under plasma action, and provided theoretical references to the mechanism of optimal regulation of plasma-induced selective apoptosis in cancer cells. It is worth noting that there are still some aspects that can be improved in this paper. For example, in the actual plasma treatment of cancer cells, the cell membrane contains a high concentration of plasma-active particles such as OH•, NO_2_^−^, NO_3_^−^, ONOO^−^, etc., and the presence of active particles will significantly change the potential difference between inside and outside of the cell and the osmotic pressure [52], so there are also differences in the concentration of the active particles between different systems. In this paper, we focus on a horizontal comparison of the transformation of transport results caused by plasma alone oxidizing hSLC19A1 before simulating the actual extracellular environment. In the next part of the study, we plan to add physiological concentrations of NACL to the extracellular environment and add OH•, NO_2_^−^, NO_3_^−^, ONOO^−^, and other active particles that play important roles in the interaction between CAP and hSLC19A1 in the extracellular system after plasma oxidation, so as to simulate the extracellular environment of the real situation, and to investigate the effect of the concentration of active particles and the resulting voltage difference on the function of hSLC19A1 to transport 5-MTHF/PMX, and the ability of the active particles to enter into cancer cells through hSLC19A1. On the prospective of the experiment based on molecular dynamics simulation, our group also plans to use plasma discharge to treat hSLC19A1 and observe the effect of plasma irradiation conditions to compare the results with those of molecular dynamics simulations from the perspective of structural biology. We also plan to conduct cellular experiments in order to study the effect of CAP on cancer cells when combined with antifolate therapy, elucidating the potentials of plasma therapy in cancer cells from the perspective of simulation in combination with experiment.

## Figures and Tables

**Table 1:** Solvent accessible surface area analysis.

Residue Type	Met	Met	Met	Met	Met	Cys	Cys	Cys	Cys
Resid Number	38	119	122	130	254	30	33	365	396
SASA	0.715	0.015	0.073	0.350	0.696	0.813	0.239	0.009	0.050

**Table 2:** Four systems of hSLC19A1/hSLC19A1_OX_ binding to 5-MTHF/PMX.

	19A1-5MTHF	19A1ox-5MTHF	19A1-PMX	19A1ox-PMX
Protein	hSLC19A1	hSLC19A1_OX_	hSLC19A1	hSLC19A1_OX_
Ligand	5-MTHF	5-MTHF	PMX	PMX

**Table 3:** Average number of hydrogen bonds present per frame for each system.

	Hydrogen Bonding Rate						Average Number of Hydrogen Bonds
OX1	Tyr281	Gln40	Tyr282	Arg133	Tyr286	Arg373	2.4197
	98.95%	53.47%	32.93%	27.24%	11.94%	10.29%	
OX2	Tyr282	Tyr281	Ser137	Gln377	Arg133	Arg157	1.7067
	70.36%	36.28%	23.54%	15.74%	7.05%	5.95%	
OX3	Tyr281	Lys411	Thr404	Glu123	Tyr282	Glu45	2.5364
	82.21%	68.67%	32.08%	27.49%	11.84%	8.30%	
OX4	Tyr282	Tyr376	Tyr281	Gln377	Arg373	Glu123	3.6851
	79.71%	78.11%	71.46%	52.62%	30.13%	21.59%	

**Table 4:** Comparison of MMPBSA calculated binding free energies.

	19A1-5MTHF	19A1ox-5MTHF	19A1-PMX	19A1ox-PMX
MM	−532.891	−570.111	−591.315	−573.037
PB	416.934	512.568	485.418	460.364
SA	−32.761	−34.905	−28.211	−27.893
COU	−331.411	−365.373	−449.946	−423.8
VDW	−201.480	−204.738	−141.369	−149.237
Tds	25.101	21.878	16.025	19.384
dG(kcal/mol)	−28.023	−16.866	−28.223	−28.963

**Table 5:** Energetic contribution of important residues to binding energy (KJ/mol).

	19A1-5MTHF	19A1ox-5MTHF	19A1-PMX	19A1ox-PMX
Cys30	0.095	0.857	0.157	2.228
Cys33	0.361	0.995	0.47	9.619
Met38	0.024	0.041	−0.184	−0.051
Arg42	−12.945	2.088	−11.012	−7.685
Glu45	25.177	15.048	11.624	7.523
Tyr126	−8.849	−7.746	−0.602	0.688
Met130	−2.145	−1.131	0.397	−3.177
Arg133	−4.388	3.084	−15.397	−16.281
Arg157	−9.451	−15.801	−11.123	−14.658
Met254	0.014	0.023	0.070	−0.078
Tyr281	−4.430	−0.925	−5.203	−2.791
Tyr282	−5.548	−9.930	−6.362	−6.548
Asp310	12.721	9.721	5.493	25.672
Arg373	−2.116	−13.064	−2.269	−0.414
Lys411	−5.756	17.921	−7.507	−9.973

## Data Availability

The data presented in this study are available on request from the corresponding author.
